# Nanopore MinION Sequencing Reveals Possible Transfer of *bla*_KPC–2_ Plasmid Across Bacterial Species in Two Healthcare Facilities

**DOI:** 10.3389/fmicb.2020.02007

**Published:** 2020-08-19

**Authors:** Catharine Prussing, Emily A. Snavely, Navjot Singh, Pascal Lapierre, Erica Lasek-Nesselquist, Kara Mitchell, Wolfgang Haas, Rita Owsiak, Elizabeth Nazarian, Kimberlee A. Musser

**Affiliations:** ^1^Wadsworth Center, New York State Department of Health, Albany, NY, United States; ^2^Maine Center for Disease Control and Prevention, Department of Health and Human Services, Augusta, ME, United States

**Keywords:** carbapenem-resistant enterobacteriaceae, *klebsiella* pneumoniae carbapenemase, horizontal gene transfer, plasmids, long-read sequencing, hybrid genome assembly, molecular epidemiology

## Abstract

Carbapenemase-producing *Enterobacteriaceae* are a major threat to global public health. *Klebsiella pneumoniae* carbapenemase (KPC) is the most commonly identified carbapenemase in the United States and is frequently found on mobile genetic elements including plasmids, which can be horizontally transmitted between bacteria of the same or different species. Here we describe the results of an epidemiological investigation of KPC-producing bacteria at two healthcare facilities. Using a combination of short-read and long-read whole-genome sequencing, we identified an identical 44 kilobase plasmid carrying the *bla*_KPC–2_ gene in four bacterial isolates belonging to three different species (*Citrobacter freundii*, *Klebsiella pneumoniae*, and *Escherichia coli*). The isolates in this investigation were collected from patients who were epidemiologically linked in a region in which KPC was uncommon, suggesting that the antibiotic resistance plasmid was transmitted between these bacterial species. This investigation highlights the importance of long-read sequencing in investigating the relatedness of bacterial plasmids, and in elucidating potential plasmid-mediated outbreaks caused by antibiotic resistant bacteria.

## Introduction

Carbapenem-resistant *Enterobacteriaceae* (CRE) are an urgent global health threat, and have been categorized by the World Health Organization (World Health Organization [WHO], 2017) and the United States Centers for Disease Control and Prevention (CDC) (Centers for Disease Control and Prevention [CDC], 2019a) as top priorities for research, drug discovery, surveillance, and control. CRE that produce carbapenemases are particularly concerning epidemiologically because carbapenemase genes can be transferred among bacteria via mobile genetic elements, including plasmids (Bonomo et al., 2017). In the United States, the most commonly identified carbapenemase is *Klebsiella pneumoniae* carbapenemase (KPC), which has become endemic in parts of the country since it was first described in 1996 (Woodworth et al., 2018; Castanheira et al., 2019).

Though first identified in *K. pneumoniae*, the gene encoding KPC has been detected across multiple genera of gram-negative bacteria (Woodworth et al., 2018; Brandt et al., 2019). Over twenty variants of *bla*_KPC_ have been described, of which *bla*_KPC–2_ and *bla*_KPC–3_ are the most frequently detected (Castanheira et al., 2019). *bla*_KPC_ genes are commonly carried inside transposons, in particular Tn*4401*, a 10kb self-mobilizing transposon in the Tn*3* family (Cuzon et al., 2011; Partridge et al., 2018). Tn*4401* and *bla*_KPC_ are often encoded on plasmids (Brandt et al., 2019), but can also be found integrated into the bacterial chromosome (Mathers et al., 2017).

Though dissemination of KPC-producing bacteria can result from epidemic spread of clonal lineages (Kitchel et al., 2009; Hargreaves et al., 2015), horizontal transfer of KPC-encoding plasmids across unrelated bacteria of the same or different species has also been described (Conlan et al., 2014, 2019; Sheppard et al., 2016; Li et al., 2018; Evans et al., 2020). Detecting such instances of plasmid transfer requires the use of whole-genome sequencing (WGS) to characterize the genomic context of antibiotic resistance genes (Boolchandani et al., 2019). As short-read sequencing has been shown to be limited in its ability to resolve the highly repetitive regions common in plasmids (Arredondo-Alonso et al., 2017), combined short-read and long-read WGS of antibiotic resistant bacteria has become an increasingly common method to better elucidate plasmid structures, and to detect plasmid-mediated outbreaks (Lemon et al., 2017; Conlan et al., 2019; Decano et al., 2019; Van Dorp et al., 2019; Wyres et al., 2019). In this study, we describe a possible case of plasmid transfer detected by hybrid analysis of short read Illumina MiSeq and long-read Oxford Nanopore Technologies (ONT) MinION sequencing, in which an identical plasmid carrying *bla*_KPC–2_ was identified across three bacterial species isolated from epidemiologically linked patients in two healthcare facilities.

## Materials and Methods

### Epidemiological Investigation

The Maine Center for Disease Control and Prevention utilizes a combination of required and voluntary reporting, along with isolate submission to the state public health laboratory (SPHL), to identify CRE that produce carbapenemases. In 2018, the SPHL identified KPC-producing bacteria in two unique clinical specimens in the same month. In response, and in accordance with guidance from the Centers for Disease Control and Prevention [CDC], 2019b, investigators arranged for colonization screens of epidemiologically linked patients who had been admitted at two associated healthcare facilities by rectal swab. Specimens from patients who had been discharged home were collected by walk-in clinics at one associated facility and by patients’ primary care providers.

The Wadsworth Center, New York State Department of Health’s public health laboratory and the Northeast Regional Laboratory for the CDC funded Antimicrobial Resistance Laboratory Network (ARLN), analyzed all rectal swab specimens from colonization screens with the Cepheid Xpert^®^ Carba-R test. This test is a real-time PCR assay for rapid detection and differentiation of five genes (*bla*_KPC_, *bla*_VIM_, *bla*_OXA–48_, *bla*_IMP–1_, and *bla*_NDM_) responsible for carbapenem resistance.

This screening identified three additional patients with KPC-producing bacteria representing three species: *Citrobacter freundii*, *Klebsiella pneumoniae*, and *Escherichia coli*. Traditionally, outbreak definitions include a requirement for matching organisms; however, as carbapenemase genes are transferrable among bacterial species and epidemiological links were present, further investigation of possible plasmid transfer was undertaken.

### Microbiological Methods

Isolates were recovered from rectal swab specimens that were positive for *bla*_KPC_ by the Cepheid Xpert^®^ Carba-R test by streaking on MacConkey agar. For clinical and colonization isolates, identification to species was confirmed by matrix-assisted laser desorption/ionization time-of-flight mass spectrometry (MALDI-TOF MS). Antibiotic susceptibility testing was performed by broth microdilution with the Thermo Scientific Sensititre Aris 2X using the GNX2F panel, and by ETEST^®^ (bioMérieux) for ceftazidime-avibactam (all specimens) and ertapenem, meropenem, and imipenem (Isolate 5 only). In addition, disk diffusion was used to test the susceptibility of Isolate 5 to doripenem, ertapenem, imipenem, and meropenem. Antibiotic susceptibilities were interpreted using CLSI M100-ED29 breakpoints for Enterobacteriales (Clinical and Laboratory Standards Institute [CLSI], 2019). The modified carbapenem inactivation method (mCIM) (Clinical and Laboratory Standards Institute [CLSI], 2019) was used to detect carbapenemase production. Molecular characterization of resistance mechanisms was performed using New York State Clinical Laboratory Evaluation Program (CLEP)-approved multiplex real-time PCR assays to detect *bla*_KPC_ and *bla*_NDM_ (developed at the Wadsworth Center), as well as *bla*_VIM_, *bla*_IMP_ (all variants), and *bla*_OXA–48__–like_ genes (developed at the CDC).

### Illumina WGS and Analysis

Genomic DNA was extracted from isolates using the DNeasy Blood & Tissue Kit on a QIAcube (QIAGEN). DNA was quantified using the Qubit dsDNA BR assay system. Sequence libraries were prepared using the Nextera XT DNA Sample Preparation Kit and sequenced on the Illumina MiSeq system at the Wadsworth Center Applied Genomic Technologies Core.

Raw Illumina reads were processed with *Trimmomatic* v0.38 (Bolger et al., 2014) and bacterial species identification was confirmed *in-silico* using *Kraken* v1.0 (Wood and Salzberg, 2014) with the MiniKraken 8GB database; paired, 250 bp reads were then de novo assembled into contigs with *SPAdes* v3.12.0 (Bankevich et al., 2012). Assembly quality was assessed using quantitative measurements, including *BUSCO* v3.1.0 (Simão et al., 2015; Waterhouse et al., 2017), prior to multilocus sequence typing analysis (MLST) with *mlst* v2.16.2^1^ and AR gene identification with *ABRicate* v0.8.13.^2^ Final analysis of the antibiotic resistance genes in the genome assembly compared gene identification between the NCBI Bacterial Antimicrobial Resistance Reference Gene Database (NCBI) (Feldgarden et al., 2019), ResFinder (Zankari et al., 2012), and Comprehensive Antibiotic Resistance Database (CARD) (Jia et al., 2016) databases to determine the best matches.

For mutation event analysis, *Mash* v1.1 (Ondov et al., 2016) was used to select the best possible reference genome from a number of candidates prior to mapping with *BWA/Samtools* [v0.7.17 and v1.9, respectively (Li and Durbin, 2009, 2010; Li et al., 2009)]. Mutation events (ME), defined as the number of single nucleotide polymorphisms (SNPs) and insertion/deletion events, were called using *FreeBayes* v1.0.2 (Garrison and Marth, 2012). A ME matrix was constructed from all isolates within a cluster by pairwise comparison of all reference-aligned sequences to count MEs while ignoring ambiguous or missing bases.

### ONT MinION WGS

For genomic DNA extraction, all isolates were sub-cultured from frozen stocks twice on blood agar plates. Colonies were resuspended in 2 ml sterile water to 4 McFarland concentration and harvested as pellets. High molecular weight genomic DNA was extracted from Isolates 1, 3, 4, and 5 using Genomic-tip 20/G (QIAGEN) and Genomic DNA buffers (QIAGEN). Genomic DNA from Isolate 2 was extracted using the Nanobind CBB Big DNA kit (Circulomics). The protocol for genomic DNA isolation for gram negative bacteria was followed for both methods as suggested by the respective manufacturer. The genomic DNA was quantified using a Qubit fluorometer (ThermoFisher Scientific). Quality of the genomic DNA was assessed using the TapeStation (Agilent).

MinION sequencing libraries were prepared from 1.5 μg of input DNA. Genomic DNA was sheared in 50 μl total volume in Covaris G tubes using an Eppendorf 5425 centrifuge at 6000 rpm. The sequencing library was prepared according to manufacturer’s instructions (Oxford Nanopore) and multiplexed using 1D Native barcoding kits (EXP-NBD104, EXP-NBD114) followed by ligation and sequencing kit (SQK-LSK109). The library was loaded on a SpotON flowcell R9.4.1 FLO-MIN106 and sequenced for 72 h on the MinION device. The fast5 data from MinKNOW was converted to fastq format using the Guppy basecaller in fast mode on a MinIT device (Oxford Nanopore, United Kingdom). The fastq reads were demultiplexed using qcat v1.0.1.^3^

### Hybrid Genome Assembly and Annotation

MinION reads were quality filtered using *filtlong* v0.2.0^4^ with a minimum read length of 1000 and the target number of bases set to 500,000,000 (to provide approximately 100X coverage of the target species’ genomes). Paired-end MiSeq reads were trimmed to remove adapters and low-quality ends (<q10) using *trim_galore* v0.6.4.^5^

Genomes were assembled using two methods: *Unicycler* v0.4.8 (Wick et al., 2017) hybrid assembly using the filtered MinION reads and trimmed Illumina reads, with default settings; and *Flye* v2.6 (Kolmogorov et al., 2019) using the filtered MinION reads, with plasmids and meta options enabled. *Flye* assemblies were polished with *racon* v1.4.7 (Vaser et al., 2017) (-m 8 -x -6 -g -8 -w 500 –no-trimming) using MinION reads mapped to the assembly with *minimap2* v2.11 (Li, 2018), followed by *medaka* v0.8.1^6^ and two rounds of *pilon* v1.23 (Walker et al., 2014) using Illumina reads mapped to the assembly with *bwa-mem* (Li, 2013). Discrepancies between the assemblies were assessed by comparing the percentage of Illumina reads that aligned to the assembly using *bwa-mem*, and the percentage of filtered MinION reads that aligned to the assembly using *minimap2*. In addition, Assembly Likelihood Evaluation (ALE) scores (Clark et al., 2013) were calculated using both the Illumina and MinION alignment files, and *Nanovar* v0.1.2 (Tham et al., 2019) was used to identify structural discrepancies between MinION reads and the assemblies. All Illumina and MinION sequences were deposited in the NCBI sequence read archive (SRA), and final assemblies in GenBank (BioProject ID PRJNA636827).

*Plasflow* v1.1 (Krawczyk et al., 2018) was used to classify contigs as plasmids or chromosomes. Final genomes were annotated with *prokka* v1.14.0 (Seemann, 2014). *Abricate* v0.9.8 (see text footnote 2) detected antimicrobial resistance genes using the CARD (Jia et al., 2016), ResFinder (Zankari et al., 2012), and NCBI (Feldgarden et al., 2019) databases, and plasmid replicon genes using the PlasmidFinder (Carattoli et al., 2014) database. Identified plasmids were queried against the PLSDB database v2019_10_07 (Galata et al., 2018) using mash (-S 42 -k 21 -s 1000) (Ondov et al., 2016) to identify related plasmids.

## Results

**FIGURE 1:**
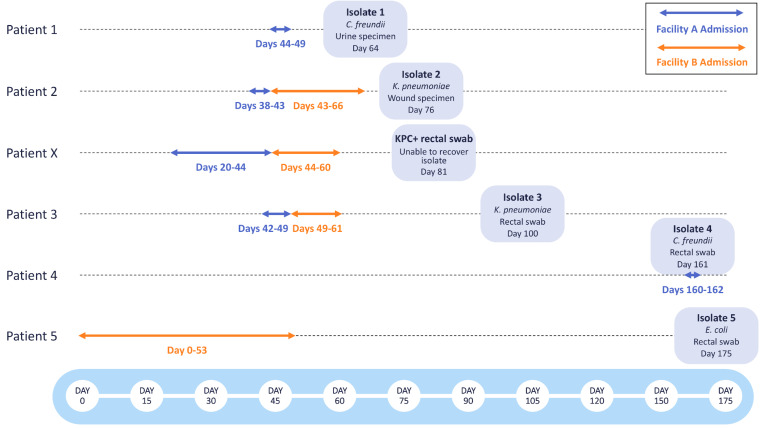
Timeline of KPC-producing bacterial isolates collected during epidemiological investigations at Facilities A and B. The admission dates of Patients 1-5 and Patient X at Facility A (blue) and Facility B (orange) are shown as arrows. The bacterial species, specimen type, and date of specimen collection are shown for each bacterial isolate.

Following the identification of two patients with KPC-producing bacteria associated with Facility A (Patient 1, Isolate 1: *C. freundii*; Patient 2, Isolate 2: *K. pneumoniae*), a retrospective investigation including colonization screening of patients who had been roommates with or who had overlapped on the same unit for three or more days with either of the two source patients was performed. Rectal swabs from two additional patients who had been on the same unit at Facility A tested positive for KPC-producing bacteria after these patients had been discharged. Isolate recovery from one rectal swab specimen was unsuccessful (Patient X), but a KPC-producing *K. pneumoniae* (Isolate 3 from Patient 3) was recovered from the second specimen. Following the initial investigation, routine point prevalence surveys were done at Facility A on the affected unit to identify any additional KPC-producing bacteria. 3 months after the initial investigation, Isolate 4 (*C. freundii*) was recovered from a rectal swab collected from Patient 4, who had recently been admitted to Facility A (Figure 1).

Prior to the initiation of this investigation, Patients 2, X, and 3 had been transferred from Facility A to Facility B. Patients 2 and X were placed in isolation at the time of admission to Facility B, but Patient 3 was assigned to a double occupancy room with a shared bathroom, sharing the room with Patient 5 for 5 days. After discharge from Facility B, a rectal swab obtained from Patient 5 yielded Isolate 5 (*E. coli*). All other rectal swabs collected from patients overlapping on the same unit for three or more days at Facility B tested negative for KPC-producing bacteria.

Isolates 1–5 differed in their antibiotic susceptibility profiles (Table 1), but all isolates were resistant to aztreonam and ticarcillin/clavulanic acid; Isolates 3 and 4 were non-susceptible to all beta-lactam antibiotics tested. Isolate 5 was susceptible to all carbapenems by broth microdilution. By disk diffusion, Isolate 5 had intermediate susceptibility to ertapenem (21 mm) and imipenem (22.5 mm), and was susceptible to doripenem (23 mm) and meropenem (23 mm). By gradient diffusion, Isolate 5 was susceptible to ertapenem (MIC 0.25 μg/ml), meropenem (MIC 0.19 μg/ml), and imipenem (MIC 1.0 μg/ml). All isolates tested positive for carbapenemase production by mCIM except for Isolate 4, which was not tested. Real-time PCR detected only *bla*_KPC_ in all isolates.

**TABLE 1:** Antibiotic susceptibility testing results.

	**Isolate 1: *C. freundii***	**Isolate 2: *K. pneumoniae***	**Isolate 3: *K. pneumoniae***	**Isolate 4: *C. freundii***	**Isolate 5: *E. coli***
**Antibiotic Class/Antibiotic**	**MIC (μg/ml)/ Interpretation**	**MIC (μg/ml)/ Interpretation**	**MIC (μg/ml)/ Interpretation**	**MIC (μg/ml)/ Interpretation**	**MIC (μg/ml)/ Interpretation**
**Aminoglycosides**					
Amikacin	≤4/S	≤4/S	≤4/S	≤4/S	≤4/S
Gentamicin	>8/R	>8/R	>8/R	>8/R	≤1/S
Tobramycin	>8/R	>8/R	>8/R	2/S	≤1/S
**Beta-lactams**					
Aztreonam	16/R	>16/R	>16/R	>16/R	16/R
Cefepime	≤2/S	8/SDD	16/R	16/R	≤2/S
Cefotaxime	8/R	>32/R	>32/R	32/R	≤1/S
Ceftazidime	2/S	>16/R	>16/R	>16/R	2/S
Ceftazidime/Avibactam	0.38/S	0.38/S	1.0/S	2/S	0.25/S
Doripenem	1/S	1/S	2/I	>2/R	0.5/S
Ertapenem	2/R	2/R	4/R	>4/R	≤0.25/S
Imipenem	2/I	4/R	4/R	8/R	≤1/S
Meropenem	4/R	2/I	4/R	8/R	≤1/S
Piperacillin/tazobactam	>64/R	>64/R	>64/R	>64/R	32/I
Ticarcillin/clavulanic acid	>128/R	>128/R	>128/R	>128/R	>128/R
**Fluoroquinolones**					
Ciprofloxacin	>2/R	2/R	>2/R	>2/R	≤0.25/S
Levofloxacin	>8/R	≤1/S	4/R	>8/R	≤1/S
**Lipopeptides**					
Colistin	0.5/NI	≤0.25/NI	≤0.25/NI	0.5/NI	≤0.25/NI
Polymyxin B	0.5/NI	0.5/NI	≤0.25/NI	0.5/NI	≤0.25/NI
**Tetracyclines**					
Doxycycline	8/I	16/R	>16/R	4/S	≤2/S
Minocycline	4/S	4/S	>16/R	4/S	≤2/S
**Glycycyclines**					
Tigecycline	0.5/NI	0.5/NI	2/NI	0.5/NI	≤0.25/NI

*Susceptibility testing was performed by gradient diffusion (Ceftazidime/Avibactam) or broth microdilution (all other antibiotics), and results were interpreted using CLSI M100-ED29 breakpoints. MIC = minimum inhibitory concentration, S = susceptible, SDD = susceptible-dose dependent, I = intermediate, R = resistant, NI = not interpretable.*

By Illumina WGS, the two *C. freundii* isolates (Isolates 1 and 4) were not able to be assigned to a sequence type, but had distinct alleles at six of seven MLST loci, and therefore were determined to be unrelated. The two *K. pneumoniae* isolates (Isolates 2 and 3) were both ST37 and were closely related, differing by only seven mutation events. Between five and twenty antibiotic resistance genes were identified from each of the five isolates (Supplementary Table 1). Two of these genes, both encoding beta-lactamases (*bla*_KPC–2_ and *bla*_TEM–1_) were identified at >90% coverage and identity from all isolates.

Hybrid genome assembly methods combining ONT MinION and Illumina reads were used to determine the location of *bla*_KPC–2_ within the genomes of the five isolates. Complete genome assemblies consisting of only circular contigs were generated for all isolates with the exception of Isolate 2, which consisted of four circular contigs and a single small (8.5 kilobase) linear contig (Supplementary Methods). Assemblies for all five isolates consisted of closed chromosomes and between two and six additional small contigs. Details on assembly evaluation and rationales for the final choice of assembly method for each isolate are provided (Supplementary Methods). Each isolate harbored at least one plasmid that carried one or more antibiotic resistance gene (Table 2).

**TABLE 2:** Characterization of plasmids carrying antibiotic resistance genes.

**Sample ID**	**Final Assembly Method**	**Plasmid ID and length (bp)**	**Plasmid replicon genes**	**Resistance genes**
Isolate 1: *C. freundii*	Unicycler hybrid	p1C157: 156,725	IncFIB(pB171), IncFII(S)	*sul1*, *arr-3*, *catB3*, *bla*_OXA–1_, *aac(6*′*)-Ib-cr*, *bla*_TEM–1_, *aac(3)-IId*, *mphA*, *sul2*
		p1C73: 73,366	None identified	*bla*_KPC–2_
		p1C44: 43,621	repA_1_pKPC-2	*bla*_KPC–2_, *bla*_TEM–1_
Isolate 2: *K. pneumoniae*	Flye	p2K157: 156,883	IncFIA(HI1)	*aac(3)-IIa*, *bla*_OXA–1_, *aac(6*′*)-Ib-cr*, *bla*_CTX–M–15_, *bla*_TEM–1_, *aph(6)-Id*, *aph(3*″*)-Ib*, *sul2*, *dfrA14*, *qnrB1*, *tet(A)*
		p2K44: 43,621	repA_1_pKPC-2	*bla*_KPC–2_, *bla*_TEM–1_
Isolate 3: *K. pneumoniae*	Flye	p3K157: 156,980	IncFIA(HI1)	*aac(3)-IIa*, *bla*_OXA–1_, *aac(6*′*)-Ib-cr*, *bla*_CTX–M–15_, *bla*_TEM–1_, *aph(6)-Id*, *aph(3*″*)-Ib*, *sul2*, *dfrA14*, *qnrB1*, *tet(A)*
		p3K44: 43,620	repA_1_pKPC-2	*bla*_KPC–2_, *bla*_TEM–1_
Isolate 4: *C. freundii*	Flye	p4C141: 140,774	IncFII(Yp), IncFIB(pB171)	*mphA*, *dfrA12*, *aadA2*, *sul1*, *sul2*
		p4C12: 12,158	Col440I_1	*bla*_KPC–2_
Isolate 5: *E. coli*	Unicycler hybrid, no depth filter	p5E44: 43,621	repA_1_pKPC-2	*bla*_KPC–2_, *bla*_TEM–1_

*Resistance genes and plasmid replicon genes with at least 90% coverage and 90% identity are shown. All contigs were circular and identified as plasmids by PlasFlow.*

Isolate 1 (*C. freundii*) carried copies of the *bla*_KPC–2_ gene on two unique plasmids: a 43,621 bp plasmid that also carried *bla*_TEM–1_ (p1C44), and a 73,366 bp plasmid that did not carry other known resistance genes (p1C73). p1C44 is 100% identical across its entire length to the plasmid pKPC_UVA01 (Genbank accession no. CP017937.1), first described in a *K. pneumoniae* isolated from an abdominal abscess at the University of Virginia Health System in 2007 (Mathers et al., 2015).

**FIGURE 2:**
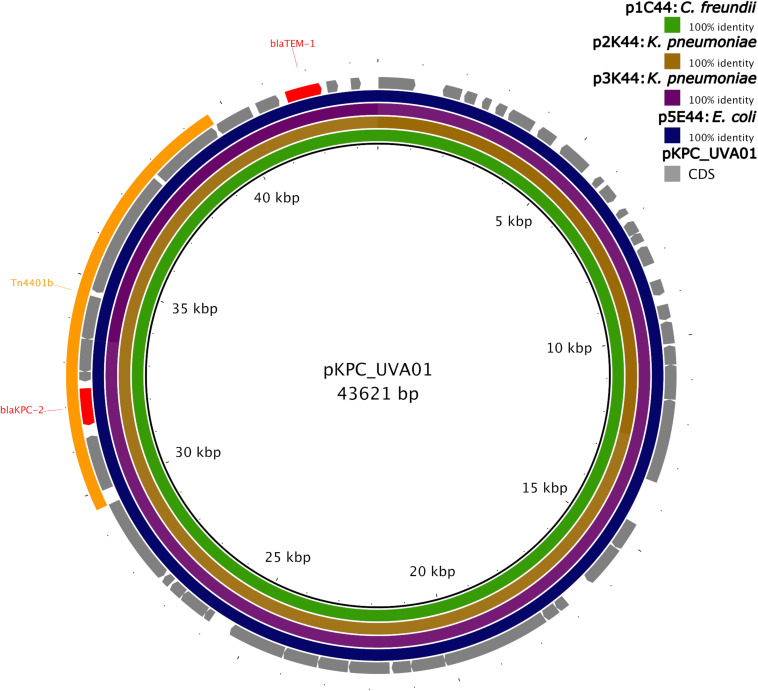
Identity of plasmids from Isolates 1, 2, 3, and 5 to pKPC_UVA01. Green, brown, purple, and blue colored circles depict the percent identity of the assembled sequence of each plasmid to the sequence of pKPC_UVA01 based on BLASTN alignment (Camacho et al., 2009). Sequences of all four plasmids were 100% identical to pKPC_UVA01 across their entire length, with the exception of p2K44, which had two single nucleotide indels compared to pKPC_UVA01, and p3K44, which had a single nucleotide deletion compared to pKPC_UVA01. The gene coding sequences (CDS) of pKPC_UVA01 are shown in gray, with resistance genes (*bla*_TEM–1_ and *bla*_KPC–2_) colored red. The location of Tn*4401*b is shown in yellow. Image created using the Blast Ring Image Generator (BRIG) (Alikhan et al., 2011).

Isolate 2 (*K. pneumoniae*), Isolate 3 (*K. pneumoniae*), and Isolate 5 (*E. coli*) also carried the *bla*_KPC–2_ gene on a plasmid identical (p5E44) or nearly identical (p2K44 and p3K44) to pKPC_UVA01 (Figure 2). Compared with pKPC_UVA01, there were two single base indels in p2K44 (a deleted thymine corresponding to position 12,990 of pKPC_UVA01 and an inserted guanine corresponding to position 30,639 of pKPC_UVA01, both located in five-nucleotide homopolymers). Similarly, there was a deleted cytosine in p3K44 in a five nucleotide homopolymer corresponding to position 13,910 of pKPC_UVA01. As the five plasmids were otherwise identical across their entire lengths, and as these indels all occurred in homopolymers, the length of which is known to be commonly mis-identified by MinION sequencers (Wick et al., 2019), they likely represent sequencing, assembly, or polishing errors and not true differences in the plasmid sequences. This is supported by mapping of Illumina reads from Isolates 2 and 3 to pKPC_UVA01, which shows that the Illumina reads do not support the deletions in p2K44 or p3K44, though they do support the insertion in p2K44 (Supplementary Methods).

**FIGURE 3:**
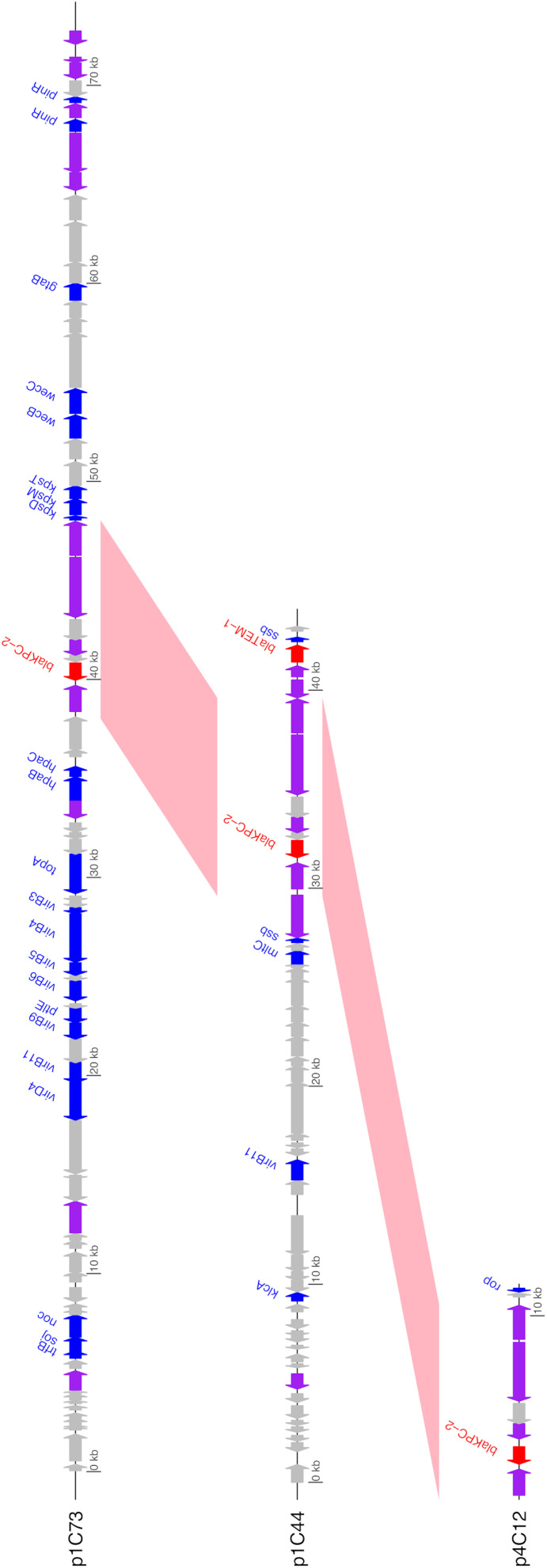
Annotated gene maps of unique identified KPC-2 plasmids. BLASTN hits from pairwise comparisons of the plasmids are shown in pink; all plasmids contain the 10 kb transposon Tn*4401b* (>99% sequence identity for both comparisons). Coding sequences (CDS) encoding resistance genes are colored in red, transposases/integrases/recombinases in purple, other identified genes in blue, and hypothetical proteins in gray. Image generated using the genoplotR package (Guy et al., 2010).

Unlike the other isolates, Isolate 4 (*C. freundii*) carried *bla*_KPC–2_ on a 12,158bp Col440I plasmid (p4C12). Like pKPC_UVA01, p1C73 and p4C12 carried *bla*_KPC–2_ within the transposon Tn4401b, but the plasmids did not otherwise share sequence identity (Figure 3). A PLSDB query found that p4C12 was most similar (mash distance 0.006, 781 of 1000 shared hashes) to a 9,803bp *bla*_KPC–3_-containing plasmid isolated from *K. pneumoniae* in Spain (GenBank accession no. NC_019151.1). No plasmids similar to p1C73 were identified (lowest mash distance 0.04, 291 of 1000 shared hashes for GenBank accession no. NZ_CP039300.1).

In addition to sharing high chromosomal genomic similarity and near-identical *bla*_KPC–2_ plasmids, Isolates 2 and 3 also shared similar 157kb IncFIA(HI1) plasmids carrying eleven antibiotic resistance genes (Table 2). Despite carrying identical replicon and resistance genes, these two plasmids were not identical across their entire sequence (mash distance 0.004, 836 of 1000 shared hashes).

## Discussion

Using a combination of short and long-read WGS, we identified identical plasmids carrying *bla*_KPC–2_ in four isolates of three bacterial genera recovered from epidemiologically linked patients associated with two healthcare facilities. The combination of these epidemiological and genetic findings strongly supports the transfer of this antibiotic resistance plasmid among different bacterial species in these facilities. Additionally, *K. pneumoniae* Isolates 2 and 3 shared high chromosomal genetic similarity, differing by only seven mutation events across their genomes. Thus, it is likely that in this case, the entire bacterial organism, including the *bla*_KPC–2_-carrying plasmid, was transferred between patients.

Isolate 4 (*C. freundii*) was identified during a routine point prevalence study at Facility A, 3 months after the outbreak. Sequencing analysis determined that this isolate was genetically unrelated to the other *C. freundii* isolate from this investigation (Isolate 1), and that the plasmid harboring *bla*_KPC–2_ in Isolate 4 was unrelated to the plasmids harboring the gene in Isolates 1, 2, 3, and 5. Further investigation of Isolate 4 identified that the patient was a resident of Massachusetts, a state that had previously identified cases of KPC-producing bacteria (Centers for Disease Control and Prevention [CDC], 2019c), and that this colonization was likely present on admission.

Upon identification of KPC-producing bacteria through colonization screening, Facility A initiated weekly rectal swabs for all patients on the affected unit. No additional KPC-producing bacteria were identified. One year later, there has been no other healthcare-onset KPC-producing bacteria identified at either Facility A or Facility B.

Prevalence of carbapenemase-producing CRE was low in Maine in 2018. At that time, most healthcare facilities in the state did not routinely conduct active surveillance cultures to identify colonized patients upon admission (Centers for Disease Control and Prevention [CDC], 2015). Facility A did note a housekeeping staffing shortage during 2018. Unrecognized colonization and missed opportunities in environmental cleaning may have played a role in the transmission of gastrointestinal flora from the source patient to other patients on the same unit in Facility A. Unrecognized colonization and a shared bathroom may have led to transmission from the source patient to the roommate at Facility B. It has been shown that toilet flushing generates aerosolized bacteria that can land on nearby surfaces or drift in air currents to land on surfaces further away, which can contribute to the direct and/or indirect transmission of gastrointestinal flora (Barker and Jones, 2005; Johnson D. L. et al., 2013; Johnson D. et al., 2013). As environmental screening was not done in the course of this investigation, it is impossible to determine if horizontal transfer of the plasmid harboring *bla*_KPC–2_ may have occurred in the environment at Facility A and/or B, or within a patient, or both.

While the epidemiological and genetic data from this investigation support the transmission of the *bla*_KPC–2_-carrying plasmid among three species of bacteria, it is also possible that the bacteria independently acquired this resistance plasmid. The identified plasmid is 100% identical to a plasmid first sequenced from *K. pneumoniae* more than 10 years earlier in Virginia (Mathers et al., 2015). This plasmid (pKPC_UVA01) has also been subsequently identified at 100% identity in other bacterial species (*Kluyvera intermedia*, *C. freundii*) from the same hospital in which it was first isolated (Sheppard et al., 2016; Barry et al., 2019), and highly similar plasmids have been identified in *Enterobacter* species from New York, Michigan, Maryland, Illinois, and Florida (Chavda et al., 2016). This suggests that, despite the fact that pKPC_UVA01 has been shown to have a relatively low conjugation efficiency into *K. pneumoniae* and *E. coli in vitro* (Hardiman et al., 2016), it may be widespread in bacterial populations. More long-read sequencing of KPC-producing bacterial populations globally is necessary to gain more insight into the phylogenetic diversity of pKPC_UVA01 and related plasmids.

The identification of three unique plasmids carrying *bla*_KPC–2_ across a small sample of KPC-producing bacteria in patients associated with two healthcare facilities highlights the diversity of the plasmid contexts of this resistance gene. The location of *bla*_KPC_ genes in transposons such as Tn*4401*b, which are themselves located on plasmids, allows for a high amount of mobility of these genes among bacterial species, patients, and healthcare facilities (Sheppard et al., 2016; Martin et al., 2017; Stoesser et al., 2017; Brandt et al., 2019; Mathers et al., 2019; Hendrickx et al., 2020). The location of *bla*_KPC_ genes in highly conserved transposons such as Tn*4401*b also highlights the necessity of using long-read sequencing to differentiate between these plasmids. Genome assembly methods based on short reads alone may not be able to assemble plasmids to the extent necessary to differentiate between plasmids that contain identical transposons on very different backbones (Figure 3). Similarly, the identification of two unique plasmids carrying *bla*_KPC–2_ within Tn*4401*b in Isolate 1 would have been impossible without the use of long-read sequencing.

Long-read and hybrid assembly of bacterial genomes is still an area of active development (De Maio et al., 2019; Wick and Holt, 2019). The discrepancies between assemblies produced by different methods in the current study, including inconsistencies in the genome assembly of Isolate 1 using Flye and in the genome assemblies of Isolates 3 and 5 using Unicycler (Supplementary Methods), support the use of more than one assembly method as well as thorough inspection and evaluation of genome assemblies.

Despite carrying a plasmid encoding *bla*_KPC–2_, and testing positive for carbapenemase production by mCIM, Isolate 5 was not carbapenem resistant by broth microdilution (Table 1) or gradient diffusion, though it showed intermediate susceptibility to ertapenem and imipenem by disk diffusion. Carbapenemase producing bacteria with low carbapenem MICs have been previously reported (Fattouh et al., 2016; Tamma et al., 2016), highlighting the importance of utilizing multiple molecular and phenotypic methods to screen clinical isolates for carbapenemase production.

The ability to identify identical plasmids across bacterial species was instrumental in defining this event as an outbreak. Genomic evidence to support the epidemiological suspicion of an outbreak was of great benefit in acceptance by the facility that an outbreak occurred and for the promotion of infection control and prevention activities needed to respond to these novel organisms statewide.

## Data Availability Statement

The datasets presented in this study can be found in online repositories. The names of the repository/repositories and accession number(s) can be found at: https://www.ncbi.nlm.nih.gov/, PRJNA636827.

## Ethics Statement

Ethical review and approval was not required for the study on human participants in accordance with the local legislation and institutional requirements. Written informed consent for participation was not required for this study in accordance with the national legislation and the institutional requirements.

## Author Contributions

CP wrote the manuscript with contributions from RO, ES, NS, and WH. RO conducted epidemiological investigation and coordinated sample collection. KM, EN, and KM supervised microbiological laboratory work. NS conducted long-read sequencing and base-calling. CP, ES, and WH conducted data analysis with guidance from PL and EL-N. All authors read and approved the final manuscript.

## Conflict of Interest

The authors declare that the research was conducted in the absence of any commercial or financial relationships that could be construed as a potential conflict of interest.

## Footnotes

1 https://github.com/tseemann/mlst

2 https://github.com/tseemann/abricate

3 https://github.com/nanoporetech/qcat

4 https://github.com/rrwick/Filtlong

5 https://github.com/FelixKrueger/TrimGalore

6 https://github.com/nanoporetech/medaka
