# Supplementary material for: Nanopore MinION Sequencing Reveals Possible Transfer of blaKPC–2 Plasmid Across Bacterial Species in Two Healthcare Facilities
Source: Front Microbiol. 2020 Aug 19;11:2007. doi: 10.3389/fmicb.2020.02007 (PMC7466660; doi:10.3389/fmicb.2020.02007)
Supplement: Supplementary file 1 [file Data_Sheet_1.PDF]

Supplementary Methods

The final assembly selected for each isolate is highlighted in bold.

Isolate 1: *Citrobacter freundii*

Table S1. Genome assembly metrics for Isolate 1.

Assembly method	**Unicycler Hybrid**	Flye
List of contigs (size in bp)	**1 (4,988,401 bp) – circular**<br>**2 (156,725 bp) – circular**<br>**3 (73,366 bp) – circular**<br>**4 (43,621 bp) – circular**<br>**5 (8,828 bp) – circular**<br>**6 (3,572 bp) – circular**<br>**7 (2,155 bp) – circular**	1/3 (4,988,768 bp) – 2 linked contigs, incomplete<br>2 (8,824 bp) – circular<br>4 (116,998 bp) – circular<br>5 (156,719 bp) – circular
Number (%) of mapped Illumina reads	**1,986,655 (99.78%)**	1,943,907 (97.63%)
ALE score (Illumina reads)	**-27,213,983**	-40,283,337
Number (%) of mapped MinION reads	**46,152 (99.95%)**	46,152 (99.95%)
ALE score (MinION reads)	**-7,991,688**	-7,429,466

Unicycler contigs 6 and 7, which were not in the Flye assembly, had very high coverage by Illumina reads but very low coverage by MinION reads, explaining the lower ALE score and higher percentage of mapped Illumina reads for the Unicycler assembly. As these two contigs were small and contained no antimicrobial resistance genes, this discrepancy was not used as a criterion for assembly selection.

Contigs 3 and 4 from the Unicycler assembly, which carried one copy of the transposon Tn*4401*b (containing *bla*$_{KPC-2}$) each, were aligned against contig 4 of the Flye assembly, which carried 2 copies of Tn*4401*b. Flye contig 4 encompassed the entire lengths of the combined Unicycler contigs 3 and 4 (Figure S1A). Nanovar [1] detected a tandem duplication in the MinION reads compared to the Flye assembly at the locations in which the plasmids were merged (positions ~73,000 and ~117,000). This discrepancy was supported by a manual inspection of the MinION read alignments, which revealed a sharp increase in coverage at the merge locations (Figure S1A). The two Unicycler contigs showed comparably smoother MinION coverage across their lengths (Figures S1B-C). The merged Flye contig was therefore determined to be an assembly error and the Unicycler assembly was selected for this isolate.

Figure S1. (A) BLASTn comparison of Unicycler contigs 3 and 4 to Flye contig 4, with the location of Tn*4401*b indicated. The mapping coverage of MinION reads to the Flye contig is shown. (B) MinION mapping coverage of Unicycler contig 3. (C) MinION mapping coverage of Unicycler contig 4. Images generated using the BLAST Ring Image Generator (BRIG) [2].

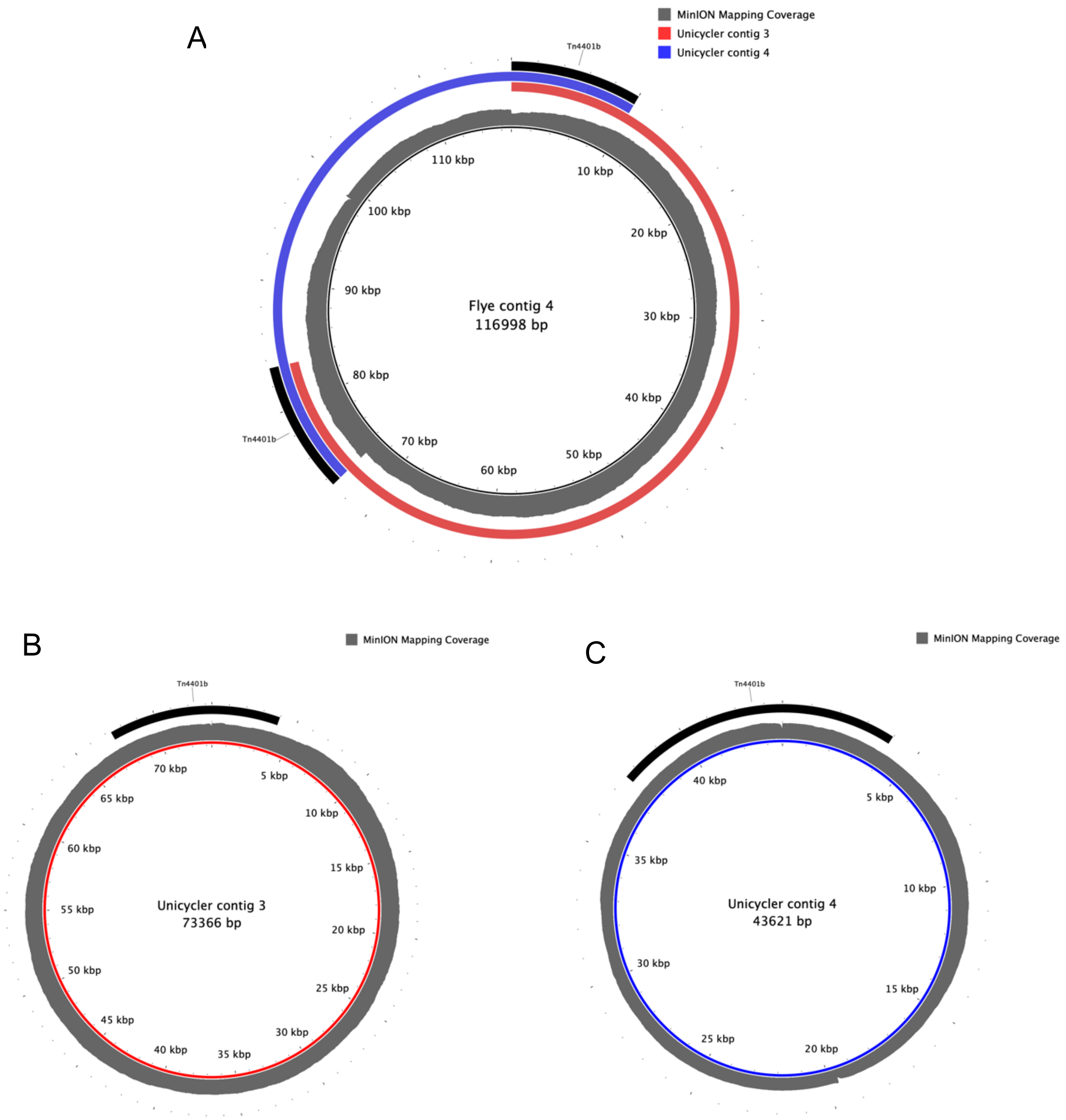

Isolate 2: *Klebsiella pneumoniae*

Table S2. Genome assembly metrics for Isolate 2.

Assembly method	Unicycler Hybrid	**Flye**
List of contigs (size in bp)	1 (5,263,116 bp) – linear<br>2 (175,689 bp) – circular<br>3 (157,606 bp) – circular<br>4 (43,622 bp) – circular<br>5 (1,167 bp) – linear<br>6 (1,091 bp) – linear<br>7 (1,077 bp) – circular	**1 (5,266,040 bp) – circular**<br>**2 (176,419 bp) – circular***<br>**3 (156,883 bp) – circular***<br>**4 (8,535 bp) – linear**<br>**5 (43,621 bp) – circular**
Number (%) of mapped Illumina reads	1,720,372 (99.46%)	**1,720,539 (99.47%)**
Number (%) of mapped MinION reads	43,830 (99.30%)	**43,831 (99.30%)**
ALE score (Illumina reads)	-47,746,630	**-48,053,717**
ALE score (MinION reads)	-97,227,773	**-94,577,837**

*These two contigs were incomplete and determined to be connected in the initial Flye assembly because they share a similar 39 kb region. As the MinION read coverage was approximately doubled in the shared 38 kb region compared to the remainder of each contig (Figure S2), and Nanovar detected breakends supporting circularization of both contigs, the contigs were split as they are believed to represent two separate plasmids.

Figure S2. Initial graph for Flye assembly of Isolate 2, showing contigs 2 and 3 joined by a shared 38 kb region and indicating the depth of each contig. Image generated using Bandage [3].

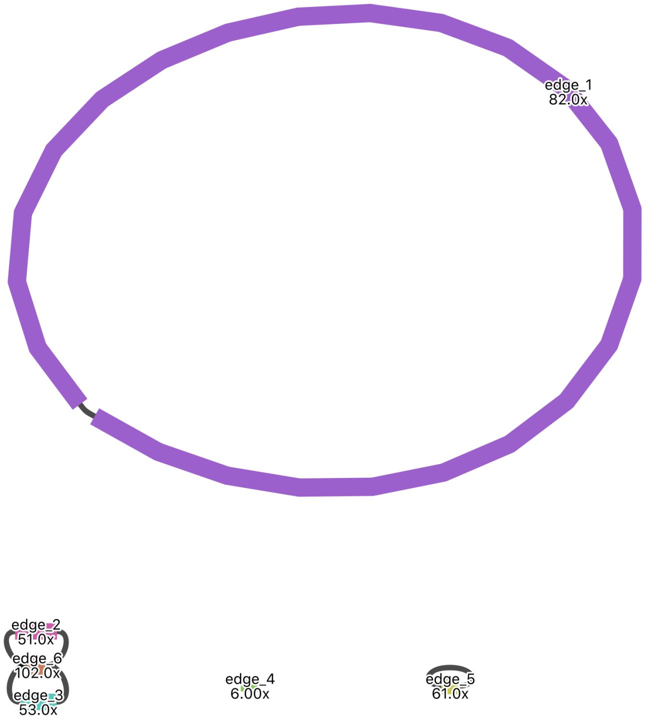

Flye contig 4, which was not in the Unicycler assembly, had very low coverage by both MinION and Illumina reads. As this contig was small, incomplete, and contained no antimicrobial resistance genes, this discrepancy was not used as a criterion for assembly selection.

The sequences of Unicycler contigs 5, 6, and 7 were 100% covered in Flye contig 1. As no structural discrepancies between the MinION reads and the Flye assembly were detected by Nanovar, and as this assembly circularized all but one contig, the Flye assembly was selected as the final assembly for Isolate 2.

The 43kb plasmid from Isolate 2 (Flye contig 5) was identical to pKPC_UVA01 (Genbank accession no. CP017937.1) except for two single base indels: a deleted thymine corresponding to position 12,990 of pKPC_UVA01 and an inserted guanine corresponding to position 30,639 of pKPC_UVA01, both located in five-nucleotide homopolymers. Mapping of Illumina reads from Isolate 2 to pKPC_UVA01 by *bwa-mem* [4] show that the deletion at position 12,990 is not supported by the Illumina reads (Figure S3), but the insertion at position 30,639 is supported by the Illumina reads (Figure S4).

Figure S3. Mapping of Isolate 2 Illumina reads to pKPC_UVA01, position 12,990, shown in IGV [5].

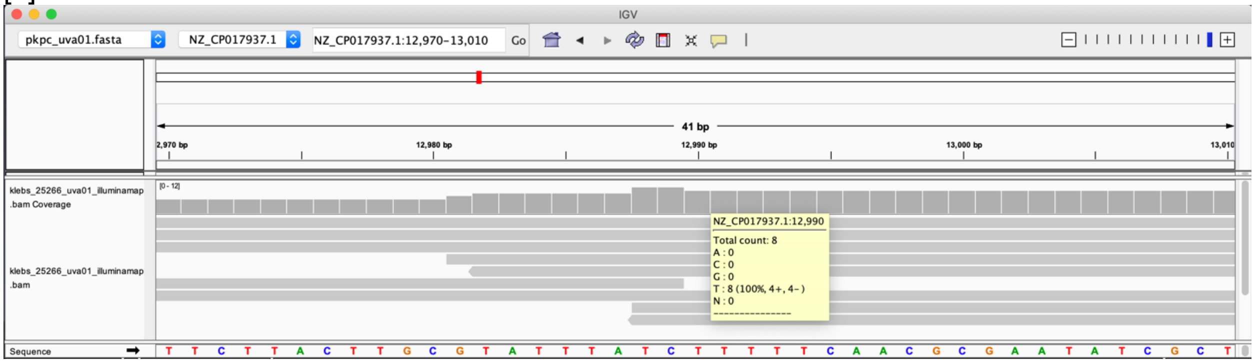

Figure S4. Mapping of Isolate 2 Illumina reads to pKPC_UVA01, position 30,639, shown in IGV.

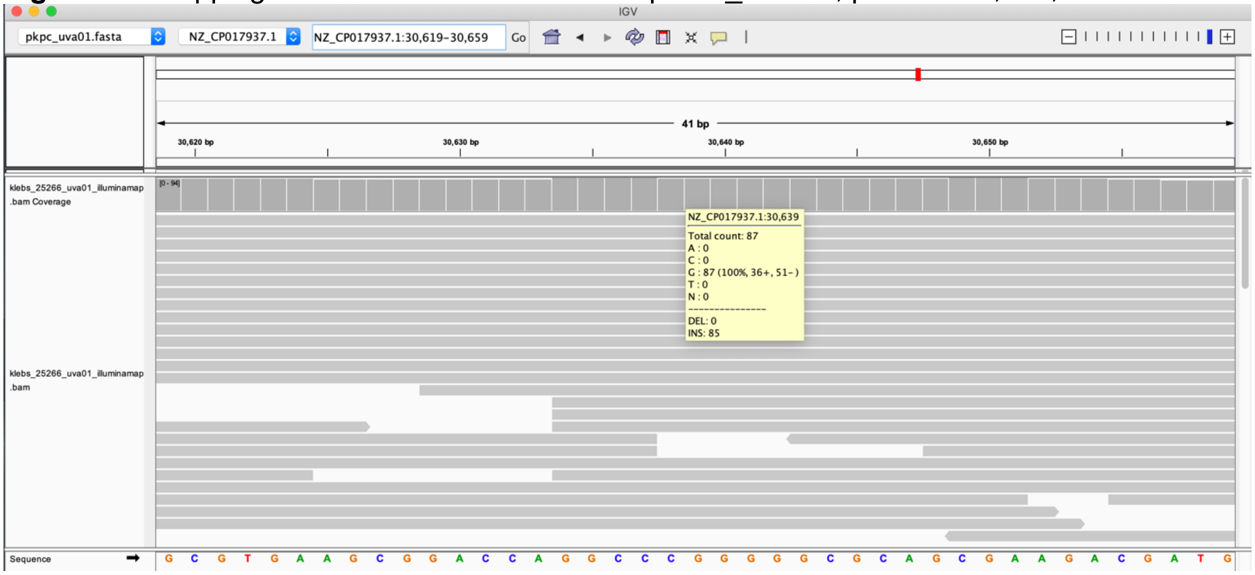

Isolate 3: *Klebsiella pneumoniae*

Table S3. Genome assembly metrics for Isolate 3.

Assembly method	Unicycler Hybrid	**Flye**
List of contigs (size in bp)	1 (5,265,871 bp) – circular<br>2 (157,181 bp) – circular<br>3 (151,107 bp) – linear<br>4 (46,597 bp) – circular<br>5 (8,297 bp) – linear	**1 (5,266,171 bp) – circular**<br>**2 (176,330 bp) – circular***<br>**3 (156,980 bp) – circular***<br>**5 (43,620 bp) – circular**
Number (%) of mapped Illumina reads	2,229,986 (99.15%)	**2,230,302 (99.17%)**
Number (%) of mapped MinION reads	45,043 (99.94%)	**45,043 (99.94%)**
ALE score (Illumina reads)	-55,055,016	**-55,346,211**
ALE score (MinION reads)	-9,977,878	**-9,314,309**

*These two contigs were incomplete and determined to be connected in the initial Flye assembly because they share a similar 39 kb region. As the MinION read coverage was doubled in the shared 39 kb region compared to the rest of each contig (Figure S5), and Nanovar detected breakends supporting circularization of both contigs, the contigs were split as they are believed to represent two separate plasmids.

Figure S5. Initial graph for Flye assembly of Isolate 3, showing contigs 2 and 3 joined by a shared 39 kb region and indicating the depth of each contig. Image generated using Bandage.

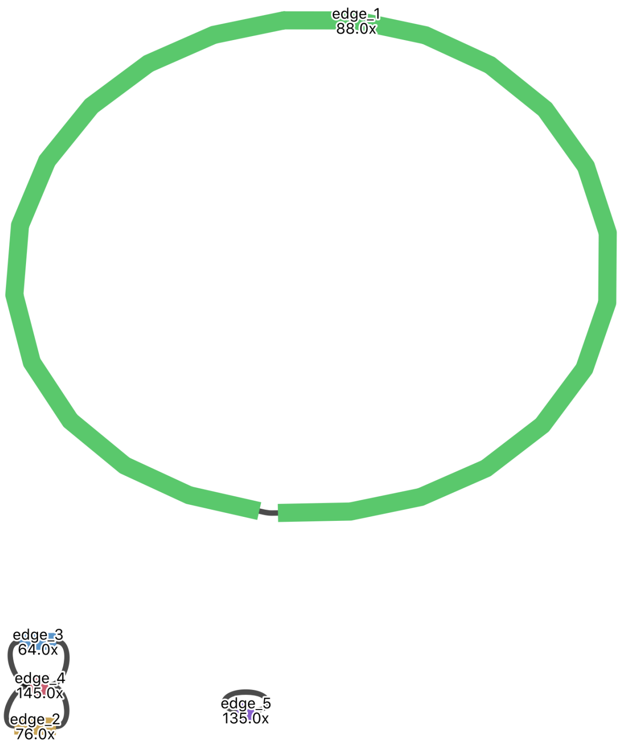

The 3kb discrepancy between the size of contig 4 in the Unicycler assembly and contig 5 in the Flye assembly (both of which contain the blaKPC-2 gene) was investigated by aligning the two contigs to determine the location of the discrepant region, then inspecting the mapping of MinION reads to the Unicycler assembly in this region (Figure S6). The discrepant 3kb region represented a gap in the MinION read alignment (position 36,901-39,873), which was also detected by Nanovar as a deletion. Therefore, the Flye assembly, which does not contain the 3kb insertion in this contig, was selected for this isolate.

Figure S6. Mapping of Illumina (top) and MinION (bottom) reads to Isolate 3 Unicycler assembly contig 4, shown in IGV.

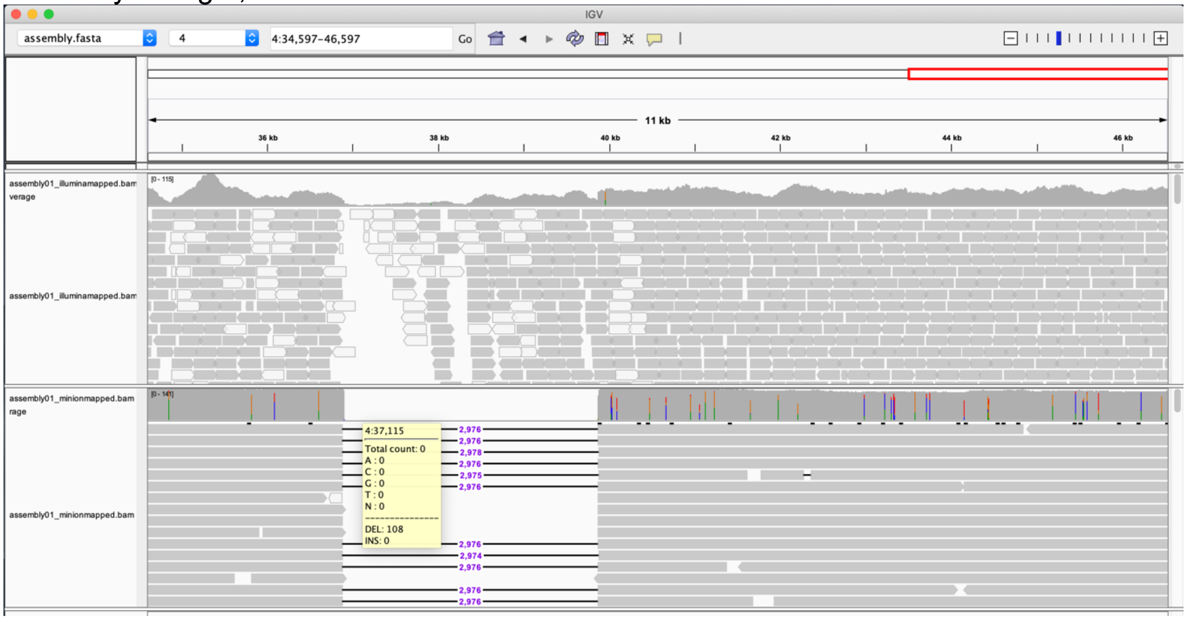

The 43kb plasmid from Isolate 3 (Flye contig 5) was identical to pKPC_UVA01 (Genbank accession no. CP017937.1) except for a deleted cytosine in a five nucleotide homopolymer corresponding to position 13,910 of pKPC_UVA01. Mapping of Illumina reads from Isolate 3 to pKPC_UVA01 by *bwa-mem* [4] show that this deletion is not supported by the Illumina reads (Figure S7).

Figure S7. Mapping of Isolate 3 Illumina reads to pKPC_UVA01, position 13,910, shown in IGV.

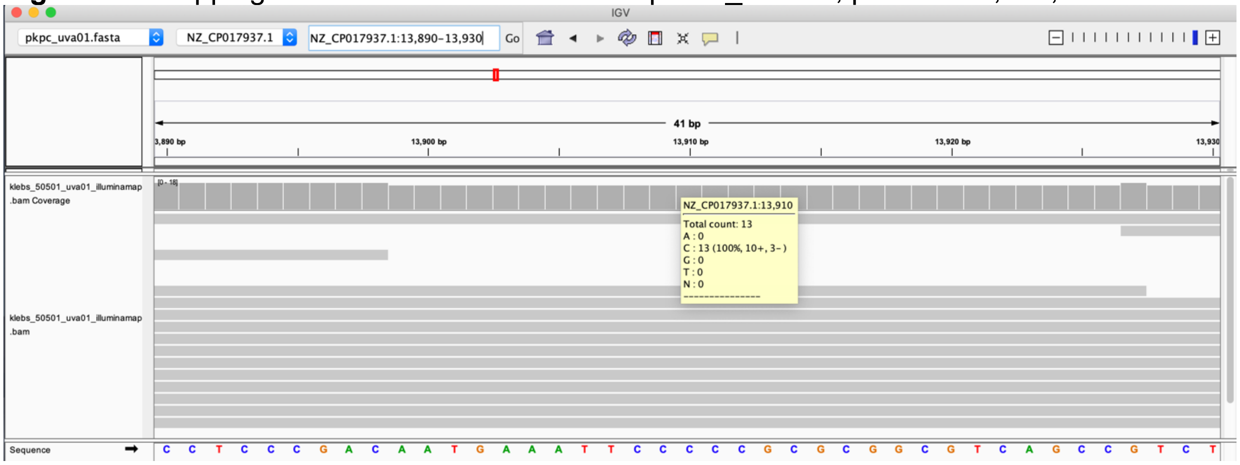

Isolate 4: *Citrobacter freundii*

Table S4. Genome assembly metrics for Isolate 4.

Assembly method	Unicycler Hybrid	**Flye**
List of contigs (size in bp)	1 (5,002,185 bp) – circular<br>2 (109,124 bp) – circular<br>3 (109,021 bp) – linear<br>4 (12,166 bp) – circular	**1 (4,995,582 bp) – circular**<br>**2 (140,774 bp) – circular**<br>**3 (109,107 bp) – circular**<br>**4 (12,158 bp) – circular**
Number (%) of mapped Illumina reads	3,623,495 (99.75%)	**3,626,885 (99.84%)**
Number (%) of mapped MinION reads	46,877 (99.94%)	**46,877 (99.94%)**
ALE score (Illumina reads)	-40,675,012	**-41,313,290**
ALE score (MinION reads)	-12,368,483	**-11,341,271**

The two assemblies were similar, but the Flye assembly was chosen due to its ability to circularize all plasmids.

Isolate 5: *Escherichia coli*

Table S5. Genome assembly metrics for Isolate 5.

Assembly method	**Unicycler Hybrid (no depth filter*)**	Flye
List of contigs (size in bp)	**1 (4,702,170 bp) – circular**<br>**2 (43,621 bp) – circular**<br>**3 (34,051bp) – circular**	1 (4,702,412 bp) – circular<br>2 (43,612 bp) – circular<br>3 (34,053 bp) – circular
Number (%) of mapped Illumina reads	**2,526,212 (99.78%)**	2,526,214 (99.78%)
Number (%) of mapped MinION reads	**42,314 (99.95%)**	42,314 (99.95%)
ALE score (Illumina reads)	**-37,982,268**	-38,502,793
ALE score (MinION reads)	**-8,320,952**	-8,194,321

*In the initial Unicycler assembly, using default settings, contig 2 (which carries the bla_{KPC-2} gene) was missing. Further investigation revealed that this was likely due to the low Illumina read coverage of this contig. The Unicycler hybrid assembly was re-run using the option –depth_filter=0, and the contig was recovered.

As the two assemblies were very similar, the Unicycler assembly was selected due to its recovery of the full shared bla_{KPC-2} plasmid (contig 2), from which 9bp were missing in the Flye assembly (contig 2).

References

1. Tham CY, Tirado-Magallanes R, Goh Y, Fullwood MJ, Koh BTH, Wang W, et al. NanoVar: Accurate Characterization of Patients' Genomic Structural Variants Using Low-Depth Nanopore Sequencing. bioRxiv. 2019; 662940. doi:10.1101/662940
2. Alikhan N-F, Petty NK, Ben Zakour NL, Beatson SA. BLAST Ring Image Generator (BRIG): simple prokaryote genome comparisons. BMC Genomics. 2011;12: 402. doi:10.1186/1471-2164-12-402
3. Wick RR, Schultz MB, Zobel J, Holt KE. Bandage: interactive visualization of de novo genome assemblies. Bioinformatics. 2015;31: 3350–3352. doi:10.1093/bioinformatics/btv383
4. Li H. Aligning sequence reads, clone sequences and assembly contigs with BWA-MEM. arXiv Prepr arXiv13033997. 2013.
5. Thorvaldsdóttir H, Robinson JT, Mesirov JP. Integrative Genomics Viewer (IGV): high-performance genomics data visualization and exploration. Brief Bioinform. 2012;14: 178–192. doi:10.1093/bib/bbs017
