# Supplementary material for: Nanopore MinION Sequencing Reveals Possible Transfer of blaKPC–2 Plasmid Across Bacterial Species in Two Healthcare Facilities
Source: Front Microbiol. 2020 Aug 19;11:2007. doi: 10.3389/fmicb.2020.02007 (PMC7466660; doi:10.3389/fmicb.2020.02007)
Supplement: Supplementary file 2 [file Table_1.pdf]

Supplementary Table 1. Antibiotic resistance genes identified from each isolate using Illumina whole genome sequencing. An X indicates that the gene was identified with >90% coverage and >90% identity, while a X* indicates that the gene was identified with lower % coverage and/or identity.

Associated Antibiotic Class	Resistance Gene	Isolate 1: *C. freundii*	Isolate 2: *K. pneumoniae*	Isolate 3: *K. pneumoniae*	Isolate 4: *C. freundii*	Isolate 5: *E. coli*
Aminoglycoside	*aac(3)-IIa*		X	X		
	aac(3)-IId	X			X	
	aadA1	X				
	aadA2				X	
	aph(3")-Ib		X	X		
	aph(6)-Id		X	X		
Aminoglycoside/ Fluoroquinolone	*aac(6')-Ib-cr*	X	X	X		
Rifamycin	*arr-3*	X				
Beta-lactam	bla_{CMY-48}	X			X	
	$bla_{CTX-M-15}$		X	X		
	bla_{EC-18}					X
	bla_{KPC-2}	X	X	X	X	X
	bla_{OXA-1}	X	X	X		
	$bla_{SHV-110}$		X	X		
	bla_{TEM-1}	X	X	X	X	X
Chloramphenicol	*catB3*	X	X*	X*		
Diaminopyrimidine	*dfrA1*	X				
	dfrA12				X	
	dfrA14		X	X		
Fosfomycin	*fosA6*		X	X		
Macrolide	*mph(A)*	X			X	
Multidrug efflux pump	*mdf(A)*	X*	X*	X*	X*	X
	oqxA		X	X		
	oqxB		X	X		
	smeE		X*	X*		
Fluoroquinolone	*qnrB1*		X	X		
	qnrB19				X*	
	qnrB81	X*				
Sulfonamide	*sul1*	X			X	
	sul2	X	X	X	X	
Tetracycline	*tet(A)*		X	X		
	tet(34)	X*	X*	X*	X*	X*
